# Reporting on genomes of endangered and threatened species supports conservation

**DOI:** 10.1093/g3journal/jkae256

**Published:** 2024-12-11

**Authors:** Lauren M McIntyre

**Affiliations:** Department of Molecular Genetics and Microbiology, University of Florida Genetics Institute, PO Box 100266, Gainesville, FL 32610, USA

Characterizing the genomes of endangered and threatened species is one of the many important tasks facing us as we attempt to understand the repercussions of the biodiversity crisis our biosphere faces. We also need to understand what we have lost and how to improve methods for recovering genomes from historical samples, including from natural history and tissue collections (Ghimire *et al.* 2024; Marino *et al.* 2023).

G3 supports these important efforts in conservation by publishing high-quality genome reports for endangered and threatened species, including the critically endangered primates the spider monkey (Pozo *et al.* 2024) and aye-aye (Versoza and Pfeifer 2024), among others. As habitat exploitation and climate change intensify, whole ecosystems are changing in response. For instance, top predators necessary for ocean health, such as the Antarctic fur seal (Hench *et al*. 2024), and once abundant pollinators, like the rusty patched bumble bee (Koch *et al*. 2023), are in decline, and species like snow algae (Hulatt *et al.* 2024; Raymond *et al.* 2024) are thriving as the ice melts, driving more rapid warming through changes in the albedo.

Capturing individual genomes is just the beginning of the work ahead. Comparative genomic analyses and understanding the differences in levels of heterozygosity between endangered species and less threatened species (Marino *et al.* 2023; Flack *et al*. 2024), as well as structural variation between species (Alexandre *et al*. 2023; Selwyn and Vollmer 2023; Guzman-Torres *et al.* 2024; Kayal *et al*. 2024), are critical for predicting evolutionary responses of populations and ecosystems as a whole to anthropogenic change (McEvoy *et al*. 2024). In addition to broad comparisons, detailed explorations of gene families can deepen our understanding, for example, in exploring resistance to pests and pathogens (Neale *et al*. 2024).

Conservation efforts depend on information about the effective population size and genetic diversity and require appropriate methods for the estimation of these parameters (Hong *et al*. 2024). Conservation efforts will depend on understanding population structures and histories. For example, understanding the impact of dramatic population declines observed in salmonids has been enhanced by such studies (Christensen *et al*. 2024). Recent genome reports on the Florida scrub Jay (Romero *et al*. 2024) and pikeminnow (Mussmann 2024) highlight the impact of fragmenting habitats on evolutionary potential.

Fortunately, the rapid pace of technological advancements, dramatic decline of reagent costs, and ongoing improvements in informatics make sequencing and annotating most genomes straightforward. Looking to the future, these advancements, combined with large-scale resource investment from governments and foundations, enable us to turn our attention to the challenges of capturing the genetic diversity, geographic context, and evolutionary history of populations. With this knowledge, conservation managers can make more informed decisions as they seek to recover and restore endangered and threatened species and the ecosystems in which they live.
